# “Metabolomic diversity of local strains of *Beauveria bassiana* (Balsamo) Vuillemin and their efficacy against the cassava mite, *Tetranychus truncatus* Ehara (Acari: Tetranychidae)”

**DOI:** 10.1371/journal.pone.0277124

**Published:** 2022-11-15

**Authors:** M. Chaithra, T. Prameeladevi, L. Prasad, Aditi Kundu, S. N. Bhagyasree, S. Subramanian, Deeba Kamil

**Affiliations:** 1 Division of Plant Pathology, ICAR-Indian Agricultural Research Institute, New Delhi, India; 2 Division of Agricultural Chemicals, ICAR-Indian Agricultural Research Institute, New Delhi, India; 3 Division of Entomology, ICAR-Indian Agricultural Research Institute, New Delhi, India; Osmania University, INDIA

## Abstract

A desirable substitute for chemical pesticides is mycopesticides. In the current investigation, rDNA-ITS (Internal transcribed spacer) and TEF (Transcriptional Elongation Factor) sequencing were used for molecular identification of six *Beauveria bassiana* strains. Both, leaf discs and potted plant bioassaye were carried out to study their pathogenicity against the cassava mite, *Tetranychus truncatus*. LC_50_ and LC_90_ values of potential *B*. *bassiana* strains were estimated. We also discovered a correlation between intraspecific *B*. *bassiana* strains pathogenicity and comprehensive metabolome profiles. Bb5, Bb6, Bb8, Bb12, Bb15, and Bb21 strains were identified as *B*. *bassiana* by sequencing of rDNA-ITS and TEF segments and sequence comparison to NCBI (National Center for Biotechnology Information) GenBank. Out of the six strains tested for pathogenicity, Bb6, Bb12, and Bb15 strains outperformed against *T*. *truncatus* with LC_50_ values 1.4×10^6^, 1.7×10^6^, and 1.4×10^6^ and with a LC_90_ values 7.3×10^7^, 1.4×10^8^, and 4.2×10^8^ conidia/ml, respectively, at 3 days after inoculation and were considered as potential strains for effective mite control. Later, Gas Chromatography-Mass Spectrometry (GC-MS) analysis of the above six *B*. *bassiana* strains was done on secondary metabolites extracted with ethyl acetate revealed that the potential *B*. *bassiana* strains (Bb6, Bb12, and Bb15) have higher levels of acaricidal such as Bis(dimethylethyl)-phenol: Bb6 (5.79%), Bb12 (6.15%), and Bb15 (4.69%). Besides, insecticidal (*n*-Hexadecanoic acid), and insect innate immunity overcoming compound (Nonadecene) were also identified; therefore, the synergistic effect of these compounds might lead toa higher pathogenicity of *B*. *bassiana* against *T*. *truncatus*. Further, these compounds also exhibited two clusters, which separate the potential and non-potential strains in the dendrogram of Thin Layer Chromatography. These results clearly demonstrated the potentiality of the *B*. *bassiana* strains against *T*. *truncatus* due to the occurrence of their bioactive volatile metabolome.

## Introduction

Red spider mites are members of the order Trombidiformes, belonging to the Tetranychidae family. Many species of this family produce silk webbing on host plants, hence they are named spider mites. The majority of species of spider mites are polyphagous [1]. The Tetranychidae is a huge family with over 900 species that can be found all over the world (Martin, 2000). *Tetranychus truncatus* Ehara (Arachnida: Tetranychidae), a spider mite species usually known as the cassava mite, was discovered on mulberry trees in Japan for the first time [2]. It has also been identified as a significant agricultural pest. This mite feeds on cassava, castor, peach, yard-long beans, papaya, peanuts, cotton, beans, eggplant, and corn [3]. Furthermore, according to [4], *T*. *truncatus* attacks on 62 host plants and its distribution is limited to Asian countries. Bionomics of *T*. *truncatus* involves creating webs by feeding on the lower surface of the leaf. *T*. *truncatus* eggs are laid beneath the webs, where the juvenile stages develop and feed on the leaves. Control of red spider mites by traditional acaricides is challenging due to their capacity to build resistance quickly. Resistance to more than 80 acaricides has been observed in its closely related species, *Tetranychus urticae* Koch (Arachnida: Tetranychidae) [5, 6]. Mites make suitable hosts for fungal related diseases as they are soft-bodied and live in humid microclimates that promote fungal growth, infection and transmission of disease [7]. There are numerous generations of mites per year as a result of high fecundity, inbreeding, arrhenotokous reproduction, and a short life cycle, especially in warmer climates. This increases resistance selection [8]. Entomopathogenic fungi (EPF) could be utilised as an alternative to synthetic pesticides to solve the pest problem and issues related to resistance [9]. Among various EPF, multiple mite species have been linked to *Beauveria bassiana* (Balsamo-Crivelli) Vuillemin (Hypocreales: Cordycipitaceae), which has been found to be particularly effective in reducing mite populations. Several experiments have also been undertaken to see whether *B*. *bassiana* could be used to manage mites biologically [10]. Several *B*. *bassiana* strains have been created as commercial biopesticides (BotaniGard^®^22 WP, Naturalis^®^TNO, Mycotrol^®^, and BotaniGard^®^ ES) for a variety of pests. In India, however, there are currently no registered formulations of *B*. *bassiana* against mites with the Central Insecticide Board and Registration Committee (CIB&RC), GOI. Therefore, the current research was carried out to identify a highly virulent indigenous *B*. *bassiana* strains against *T*. *truncatus*.

The generation of pathogenic enzymes and secondary metabolites is known to occur in Biological Control Agents (BCAs). Secondary metabolites are genetic traits inherent in an organism that are commonly used by fungi to adapt to their surroundings [11]. Metabolomics is a high-throughput technology for studying secondary metabolites that has the potential to provide unique insights into biological processes involving small molecules. The few metabolites that have received extensive research are destruxins formed by *Metarhizium* spp. Sorokin (Hypocreales: Clavicipitaceae) [12], bassianolide and beauvericin are secreted by *B*. *bassiana* [13, 14], and hirsutellin is produced by *Hirsutella thompsonii* Fisher (Hypocreales: Ophiocardicipitaceae) [15]. The amount of these secondary metabolites produced differs by genus, species, and growth conditions. Several studies on the virulence of *B*. *bassiana* on insect hosts have been undertaken [16]. However, few investigations have been conducted to characterize the variance in metabolite profiles across strains of specific fungal BCAs. Hence, the current work attempts to evaluate the diversity in metabolite profiles among six *B*. *bassiana* strains produced in identical conditions to correlate with their pathogenicity.

The EPF link to cassava mites is poorly understood. In our previous article, we reported the primary screening for the virulence of different strains of *B*. *bassiana* collected from various sources and geographical locations against *T*. *truncatus* (unpublished). Out of 30 strains, we chose 3 virulent (Bb6, Bb12 and Bb15) and 3 non-virulent (Bb5, Bb8, and Bb21) strains for the current investigation to establish a dose response relationship between the concentration of potential *B*. *bassiana* strains and the *T*. *truncatus* mortality rate. Furthermore, a correlate between the potentiality of *B*. *bassiana* strains against *T*. *truncatus* and their metabolite profiling patterns as determined by Gas Chromatography Mass Spectrometry (GCMS) and Thin Layer Chromatography (TLC) analysis was discussed.

## Materials and methods

### Mite culture

Isofemale lines of *T*. *truncatus* culture were raised on clean mulberry (*Morus alba* Linnaeus (Rosales: Moraceae)) leaves kept on sterilized foam sheets saturated in water in an insect- proof climate control chamber at 27±1°C, 12:12 L:D photoperiod, and 65±5% relative humidity. The bioassay was performed on newly emerged one or two days old females.

### Identification of *B*. *bassiana*

#### Morphological identification of *B*. *bassiana*

The six strains of *B*. *bassiana* under investigation were isolated from the soil and body of an insect cadaver. Under a compound microscope, the fungus was morphologically identified and micro-morphological observations, photomicrographs, and measurements were made using the camera (Progres capture pro2.7- JENOPTIK) attached to the microscope. Humber’s identification key was used to identify all the strains [17].

#### Molecular identification of *B*. *bassiana*

The fungal DNA of each strain of *B*. *bassiana* was extracted from seven-day-old cultures using the CTAB technique [18]. A pairs of Internal Transcribed Spacer (ITS) and Elongation Factor 1-α (EF1-α) primers [ITS1: 5’- TCGGTAGGTGAACCTGCGG-3’, ITS4: 5’- TCCTCCGCTTATTGATATGC-3’ [19]; EF1-983F: 5’- GCYCCYGGHCAYCGTGAYTTYAT-3’, EF1-2218R: 5’- ATGACACCRACRGCRACRGTYT-3’ [20], were used to amplify the fragment ITS and EF1-α region. The amplification reaction conditions were followed exactly as reported (ITS and EF1-α—[21]). The PCR results were run on a 1.8% agarose gel and sequenced using the Sanger sequencing technique by outsourcing (Anuvanshiki (OPC) Pvt. Ltd). After sequencing, Maximum Likelihood (ML) was used to build phylogenetic trees, and the Jukes-Cantor technique with 1000 bootstrap replicates was used to determine the topology of the trees. MEGAX -Molecular Evolutionary Genetics Analysis-conductedphylogenetic analysis.

### Preparation of the conidial suspension

Six selected *B*. *bassiana* strains and one strain from a commercial formulation (Beveroz: *B*. *bassiana*, Utkarsh®) were grown for 7–10 days on PDA medium plates at 25±2°C until extensive conidiation occurred. Conidia were collected from cultures that had been growing for two weeks by flooding the plates with sterile aqueous (0.05%) Tween-80. To get rid of any mycelial pieces, the end product was filtered through sterile muslin fabric. The suspensions were subsequently adjusted to desired concentrations (1×10^5^, 1×10^6^, 1×10^7^, 1×10^8^ and 1×10^9^) conidia/ml using an improved Neubauer haemocytometer (HBG, Germany) under a compound microscope, depending on the counts of conidia in 1mL of suspensions (40× magnifications). The viability of each strain was determined by germinating conidia on PDA medium. As described by [22], the strains were suspended in 0.01% Tween-80 at 10^5^ conidial concentration in 100 μL. The germ tube produced from a conidium was regarded as have germinated if it was at least as long as the conidium’s width.

### Bioactivity of *B*. *bassiana*

#### *In-vitro* screening of *B*. *bassiana*

In November 2021, the efficacy of *B*. *bassiana* strains against *T*. *truncatus* was tested in the laboratory. Mulberry leaves were chopped into 4 cm^2^ discs and both sides of the chopped leaves were sprayed with 1 mL of six collected and one commercial strain of *B*. *bassiana* solution (1×10^8^ conidia per mL) using a Potters tower (Burkard manufacturing Co Ltd.). After drying, the leaf discs were placed upside down in 11.0 cm disposable Petri dishes on half inch damp white foam sheets. On the leaf disc of each plate, 30 mites were released. Each strain was replicated five times. As a control, water containing 0.02% Tween-80 was sprayed on both sides of the leaf discs. The mortality was recorded at intervals of 24 hours, and mites were deemed dead if their appendages did not move when touched with a fine brush. The death mites were put on autoclaved Petri plates with a moist filter paper lining in order to promote *B*. *bassiana* growth on the surface of the mite’s cadavers. Mortality of *T*. *truncatus* caused by *B*. *bassiana* was viewed by microscopic examination of spores on the mite’s surface. The same experiment was repeated twice with the same set of conditions.

#### Potted bean plant assay

*In-vitro* screening of six collected and one commercial *B*. *bassiana* strain against *T*. *truncatus* was further confirmed by green house studies. The 1×10^8^ conidia mL^-1^ of conidial concentration was used in this experiment twenty *T*. *truncatus* females were introduced to each 20 days old kidney bean plant and allowed to establish for 10 days. Five replications were maintained for each treatment. An initial observation of adult cassava mites was done on the day before the first treatment, and continued for ten days. Mite-infested kidney bean plants were sprayed with a *B*. *bassiana* spore suspension using hand automizer, while the control treatment received simple distilled water. Five leaves in each plant were examined for the efficacy of *B*. *bassiana* application based on the observations of the survivability of adult spider mites for every 48 HAS (hours after spray) interval using a hand magnifier.

#### Dose response bioassay

Three *B*. *bassiana* strains showed severe sporulation, causing higher mortality and mycosis rates in *in-vitro* screening, and the potted bean plant assay was further evaluated at four different doses (1×10^6^, 1×10^7^, 1×10^8^ and 1×10^9^ mL^-1^) of conidia. For each strain, conidial suspensions were prepared in a 0.02% Tween-80 solution and sprayed on the mites as mentioned above. As a negative control, 0.02% Tween-80 solution alone was also used. Prior to application, the viability of the conidia was calculated.

### Production and extraction of secondary metabolites

*B*. *bassiana* strains were cultured in PDB for 14 days, then the culture filtrates were collected and the pH was reset with 37 percent (wt/vol) HCl to 2.0. The metabolites were extracted three times using the same volume of ethyl acetate, and the combined ethyl acetate extracts from the three biological replicates were then dried with a rotary evaporator (Heidolph, Germany) and re-suspended in HPLC grade methanol (1 mL). The extracts were dried over anahydrous Na_2_SO_4_ and evaporated under vacuum at 60°C to concentrate the metabolites. The metabolites were then dissolved in HPLC grade methanol and analysed using gas chromatography-mass spectrometry (GC-MS) [23].

### GC-MS analysis

Analytes were dissolved in GC-MS grade ethyl acetate (2 mL) separately and filtered with a 0.45 μm membrane (Millipore, Billerica, MA). Each sample was analysed with a 5590C Agilent GC-MS (Agilent Technologies^®^, USA) to identify the volatile organic components. Components were separated through an Agilent HP-5MS column (30m × 0.25 mm, film thickness 0.25μm) and detected by the mass spectrometer. Helium gas (> 99.99% purity) was used as a carrier gas with a flow rate of 1.0 mL min^-1^ and a pressure of 10 psi. Each sample (1 μL) was injected into GC through an in-built auto-injector with the split ratio of 20:1. A GC-MS temperature programme was developed which started with 40°C and enhanced at a rate of 3°C min^-1^ to reach 130°C, then held for 1 min. Further, the temperature increased at a rate of 5°C min^-1^ to reach 210°C. Then the temperature was increased at the rate of 10°C min^-1^ to reach 300°C. The total run time was 56 min. The MS acquirement parameters were programmed with the 200°C temperature of ion source, 70 eV electron ionization, transfer line temperature of 200°C, full scan mode (50–550 AMU), solvent delay of 2 min, and 1214 V E.M. voltage. Volatile organic components were characterized by matching with NIST mass spectral library mass spectral data, corresponding retention index (RI), and mass fragmentation pattern [24].

### TLC analysis

Thin Layer Chromatography was carried out on commercial silica gel-H TLC plates (chloride-0.02%, sulphate-0.02%, iron-0.02%, heavy metals-0.02%, and pH-7) for principal component partition. The capillary tubes were used to spot 6 metabolite extracts of *B*.*bassiana* strains at one inch above the bottom of the plate. Then, as mobile phases for TLC, we devised a variety of solvent solutions because different solvent systems may separate biological molecules, Toluene, ethyl acetate, and formic acid were used as mobile phase solvents in various ratios (5:4:1). After the running procedure, the plates were viewed under UV light at 350 nm.

### Data analysis

The results were expressed as percentages (%). Percentage mortality was corrected according to Abbott [25]. The arc-sin transformation was used to transform the corrected mortality, which was then analysed using analysis of variance (ANOVA). Duncan’s Multiple Range Test (DMRT) was used to compare the percentage mortality rates [26]. In the POLO-PC package software, LC50 and LC90 values were calculated in accordance with Finney [27]. Using IBM SPSS 23.0, all statistical analyses were carried out [28]. Principal component analysis (PCA), heatmap creation, and hierarchical clustering were used to statistically examine GC-MS data using JMP software (version 14) [29]. The % area values were employed as independent variables in this multivariate analysis. Similarly to TLC, the multivariate analysis of similarity or dissimilarity between the metabolite profiles of all *B*. *bassiana* strains was computed using the NTSYSpc-2.20e edition (Numerical Taxonomy System Programme) software [30].

## Results

**Fig 1:**
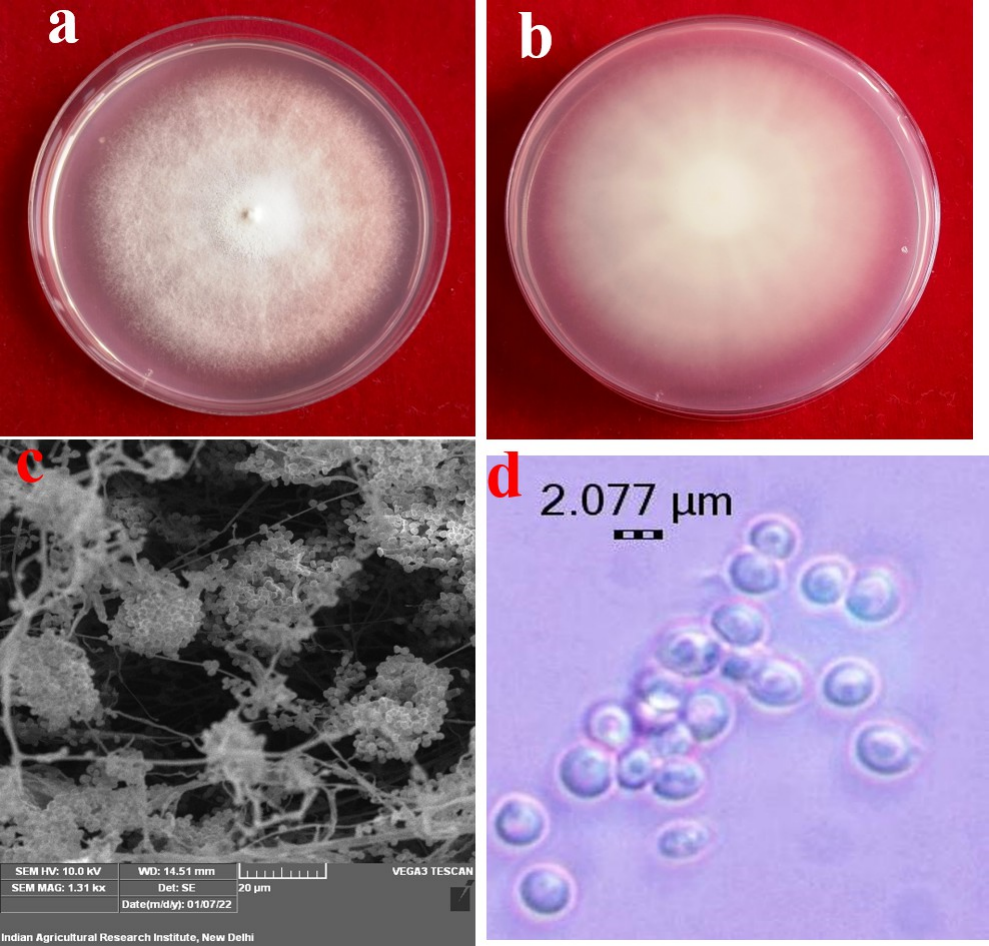
Morphological observations of *B*. *bassiana*. Colony appearance on PDA a) front view; b) reverse view; c) conidial cluster under SEM; d) conidia.

**Fig 2:**
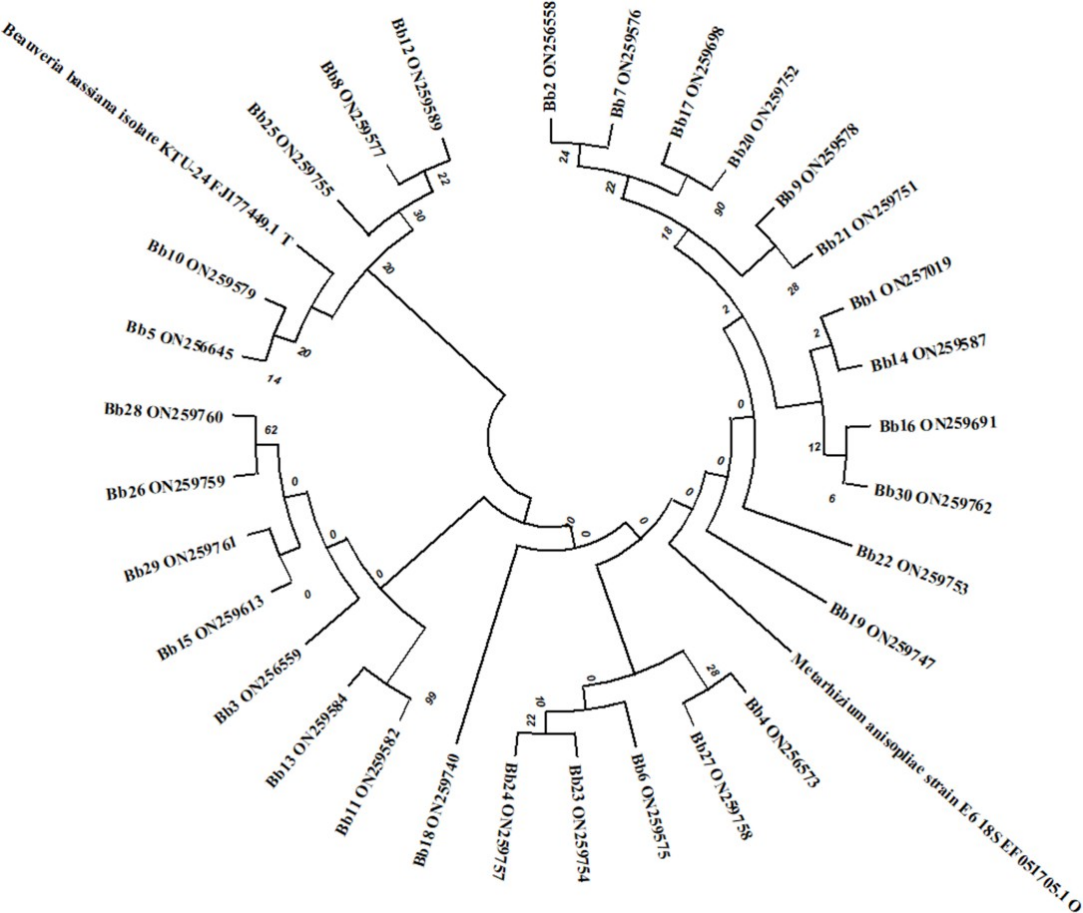
Phylogenetic tree of constructed by Maximum Likelihood method and relationship showing by Jukes-Cantor method between the ITS regions of six strains of *B*. *bassiana* and related species. The "bootstrap" values for 1,000 repetitions are indicated next to the tree branches.

**Fig 3:**
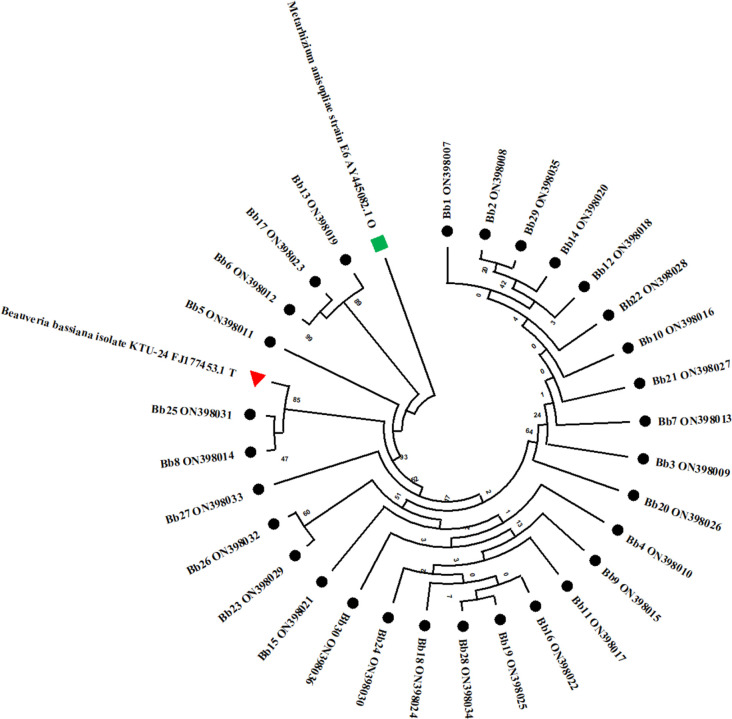
Phylogenetic tree of constructed by Maximum Likelihood method and relationship showing by Jukes-Cantor method between the TEF regions of six strains of *B*. *bassiana* and related species. The "bootstrap" values for 1,000 repetitions are indicated next to the tree branches.

The six collected *Beauveria* strains (Bb5, Bb6, Bb8, Bb12, Bb15 and Bb21) were identified as *B*. *bassiana* using Humber’s identification key based on morphological characteristics, *i*.*e*., colony appearance, size and shape of the conidia (Fig 1). As part of molecular identification, the ITS1- 5.8 s-ITS2 and TEF regions of 6 *B*. *bassiana* strains were amplified using ITS1 and ITS4, as well as EF1-983F and EF1-2218R primers, respectively. After the PCR products were sequenced, the rDNA homology was checked using the BLAST (Basic Local Alignment Search Tool) programme through the NCBI database, which confirmed all these strains as *B*. *bassiana*, and the sequences were submitted to GenBank. These six *B*. *bassiana* strains taxonomic identity was established by the alignments and phylogenetic analysis (Figs 2 and 3).

### *In vitro* leaf disc bioassay

**Fig 4:**
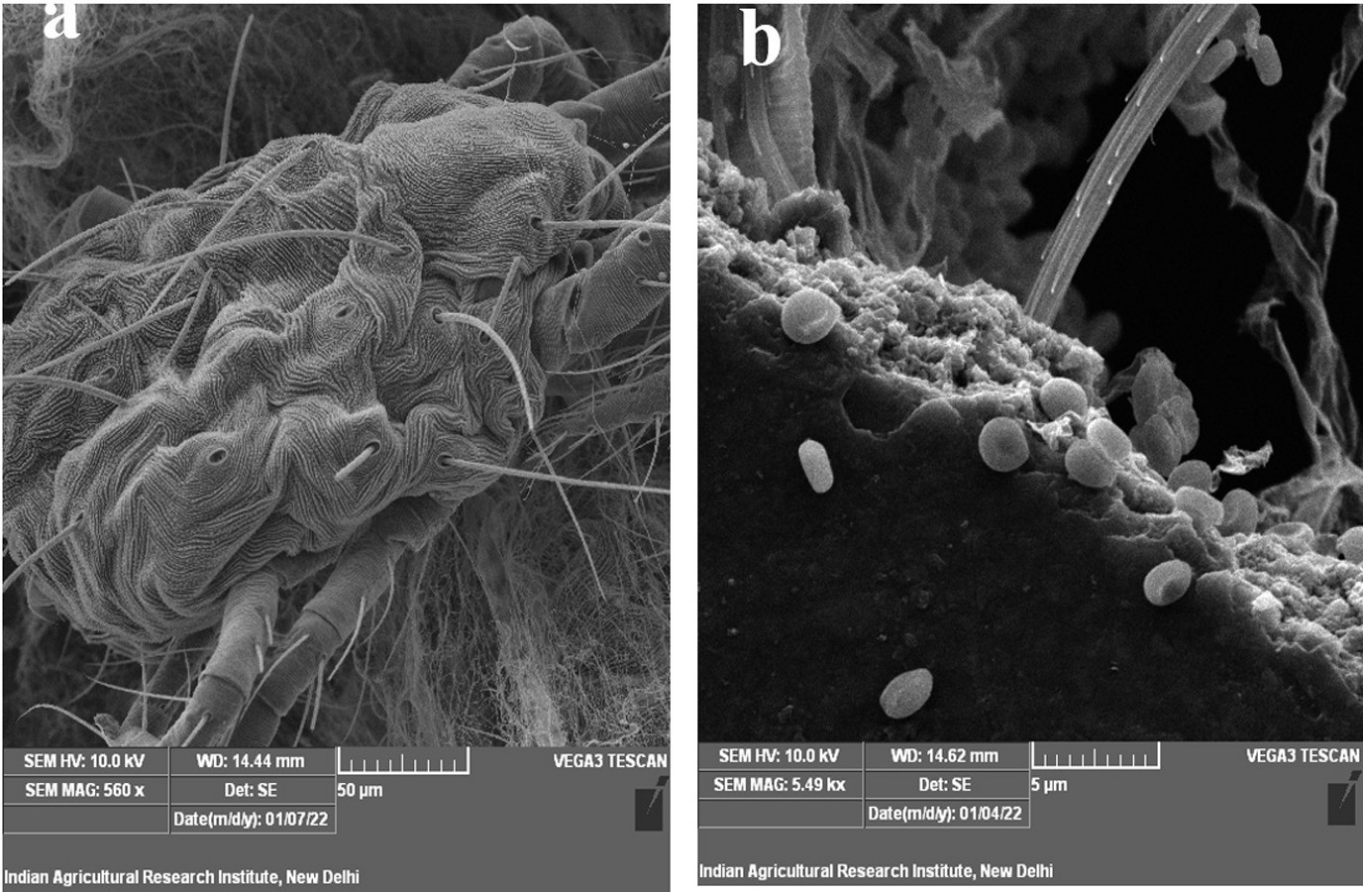
Confirmation of death of mite due to *B*. *bassiana*. a) Normal death (no mycelia and spores); b) Death due to *B*. *bassiana* (mycelia and spores are seen on mite cadaver).

Three days after application of the conidial suspension of *B*. *bassiana*, the death of mites due to mycosis was observed. Before dying, infected female mites become sluggish, darker, and slightly enlarged. Many of the dead mites were well mycotized under high RH conditions (Fig 4). No mycotic cadavers were observed in control dead mites (Fig 4). Out of the six *B*.*bassiana* strains tested, Bb6, Bb12, and Bb15 showed significantly higher rates of mortality of *T*. *truncatus* than the commercial strain Beveroz (67.30%) at 3 DAI (S1 Table). But Bb5, Bb8, and Bb21 showed the lower rates of mortality against *T*. *truncatus*, with respective rates of mortality of 6.49%, 5.02%, and 8.71%. Similar to this, at 5 DAI, the amount of cadavers that supported sporulating mycelium (mycosis rate) was significantly higher for Bb6, Bb12, and Bb15 strains (85%, 84%, and 88%, respectively) and lower for Bb5, Bb8, and Bb21 strains (0%, 1%, and 1%, respectively) (S1 Table).

### Potted bioassay

The efficacy of six *B*. *bassiana* strains against *T*. *truncatus* populations on potted bean plants was tested. The initial mean population of *T*. *truncatus* per plant was 94.80, 102.80, 79.00, 100.80, 90.60, and 85.60 before spraying of Bb5, Bb6, Bb8, Bb12, Bb15, and Bb21 conidial suspension, (Table 1). In the case of Bb6, Bb12, and Bb15 strains, the number of mites was reduced after *B*. *bassiana* application from 48 to 72 HAS. On the other hand, the potted bean plants sprayed with Bb5, Bb8, and Bb21 sttrains exhibited an increase in *T*. *truncatus* population from 48 to 72 HAS. repetitive measures ANOVA on 48 to 96 HAS revealed significant differences in the *T*. *truncatus* survibility among *B*. *bassiana* strains (Table 1). In the control, the *T*. *truncatus* population increased rapidly, with an accomplishment of about 114.20, 137.00 and 150.00 per plant after 48, 78 and 96 HAS, respectively, and the mite-infested leaves turned white and withered. However, out of the 6 strains, 3 strains (Bb6, Bb12 and Bb15) showed the highest potentiality against *T*. *truncatus* based on laboratory and greenhouse bioassay tests. Therefore, only these three potential strains were used in the multiple dose bioassay experiment and the other three strains (Bb5, Bb8 and Bb21) were considered non-potential against *T*. *truncatus*.

**Table 1:** Evaluation of potential and non-potential *B*. *bassiana* against *T*. *truncatus*.

Strains		No. of *Tetranychus truncatus* / 25 leaves		
	Before spray	48 HAS	72 HAS	96 HAS
Bb5	94.80 ± 12.62abc	90.80 ±12.32d	101.00 ± 13.10b	111.40 ± 13.89b
Bb6	102.80 ± 8.26c	61.80 ±12.24ab	38.20 ± 8.17a	24.20 ± 5.45a
Bb8	79.00 ± 15.64a	74.80 ± 12.52bcd	85.00 ± 6.60b	98.40 ± 6.27b
Bb12	100.80 ± 8.56bc	70.60 ± 13.01abc	53.40 ± 4.16a	31.20 ± 4.21a
Bb15	90.60 ± 7.33abc	57.20 ± 2.59a	43.00 ± 7.14a	24.80 ± 3.96a
Bb21	85.60 ± 14.84ab	80.00 ± 16.40cd	91.20 ± 21.58b	106.60 ± 20.33b
Control	104.00 ± 8.46c	114.20 ± 11.64e	137.00 ± 18.22c	150.00 ± 7.25c
F ratio	3.48	12.92	38.86	118.42
df	34	34	34	34
p value	0.0214	0.000001	0.000001	0.000001

*Means followed by the same letter within the same column are not significantly different (p<0.05).

### Multiple-dose bioassay of *B*. *bassiana* strains with high potential

The adult mortality of *T*. *truncatus* in response to various conidial concentrations of three potential *B*. *bassiana* strains is shown in Table 2. In this investigation, the LC_50_ and LC_90_ values of *B*. *bassiana* strains were calculated using probit analysis. The LC_50_ values of Bb6 and Bb15 against *T*. *truncatus* populations after 3 DAI were the same, *viz*., 1.4×10^6^ conidia/mL, whereas for Bb12 strain was 1.7×10^6^ conidia/mL. However, Bb6 and Bb15 LC_50_ values were lower than Bb12. (Table 2). At 3 DAI, the LC_90_ values of strains Bb6, Bb12, and Bb15 to *T*. *truncatus* populations were 7.3×10^7^, 1.4×10^8^, and 4.2×10^8^ conidia/mL respectively. The morality of *T*. *truncatus* significantly increased when conidial concentrations *B*. *bassiana* strains increased, being highest at concentrations of 1×10^8^ and 1×10^9^. There was no significant variation in LC50 values of all *B*.*bassiana* strains at 1×10^8^ and 1×10^9^ conidia/mL concentrations.

**Table 2:** Median lethal concentration (LC_50_ and LC_90_) of *B*. *bassiana* strains against *T*. *truncatus* adults.

Strains	LC_50_ (conidia/mL)^a^	(LC_50_) 95% confidence interval	LC_90_ (conidia/mL) ^b^	(LC_90_) 95% confidence interval	Slope±SE^c^	Heterogeneity	^d^X^2^	df	p value
Bb6	1.4×10^6^	0.3×10^5^–5.1×10^6^	7.3×10^7^	2.0×10^7^–4.0×10^9^	0.750±0.073	2.25	4.51	2	0.1049
Bb15	1.7×10^6^	0.4×10^5^–5.9×10^6^	1.4×10^8^	3.7×10^7^−3.1×10^9^	0.660±0.070	1.83	3.67	2	0.1596
Bb12	1.4×10^6^	5.7×10^5^−2.7×10^6^	4.2×10^8^	1.8×10^8^–1.5×10^9^	0.519±0.066	0.30	0.59	2	0.7445

^a^LC_50_ Concentration (conidia mL−1) at which 50% mortality observed.

^b^LC_90_ Concentration (conidia mL−1) at which 90% mortality observed

^c^Slope at the response of regression equation ± standard error.

^d^X^2^, Chi-squared values at different df and probability level (0.05).

### Metabolite profiling of potential and nonpotential *B*. *bassiana* strains

#### Volatile organic compounds as analysed in GC-MS

Six *B*. *bassiana* strains culture filtrates were extracted with ethyl acetate, and GC-MS analysis was used to assess diversity of metabolomics profiles. The present experiment observed the intraspecific variation among the identified metabolites of six *B*. *bassiana* strains. Highly potential strains Bb6, Bb12 and Bb15 were found to contain 13 (S2 Fig), 12 (S4 Fig) and 12 (S5 Fig) volatile metabolites respectively (Table 3), representing alkanes, alkenes, carboxylic acid derivatives, alcohol, unsaturated fatty acids, and hexadecanoic acid derivatives. In non-potential strains (Bb5, Bb8 and Bb21), 15 (S1 Fig), 15 (S3 Fig), and 12 (S6 Fig) volatile metabolites respectively (Table 3), representing alkanes, alkenes, carboxylic acid derivatives, alcohol, unsaturated fatty acids, and hexadecanoic acid derivatives. In non-potential strains (Bb5, Bb8 and Bb21), 15, 15, and 12 volatile metabolites respectively were found (Table 3). Interestingly, the number of volatile metabolites was higher in non-potential strains, but the probable acaricidal metabolites were found in higher abundance in potential strains (Table 3). Higher concentration of Bis (dimethylethyl)-phenol *i*.*e*., 6.15%, 5.79% and 4.69% produced in potential strains *i*.*e*., Bb12, Bb6 and Bb15 respectively. Apart from its acaricidal activity, Bis (dimethylethyl)-Phenol also has repellent, and oviposition deterrent properties [31]. Insect derived plant regulators, *i*.*e*., Docosene [32], were detected in corresponding strains of Bb8 (3.54%), Bb12 (8.81%), Bb15 (4.30%) and Bb21 (7.90%). Among these strains, Bb12 produced a higher amount of long chain hydrocarbons, which might have helped penetrate the outer coverage of mites. Similarly, potential strains, Bb6 and Bb12 produced 19.51% and 4.72% of Nonadecene compounds, which have defense secretion functions, helping to overcome insect innate immunity mechanisms. However, Nonadecenewas not detected in other strains except for Bb8 (5.64%). All six strains contained Octadecene, Hexadecene, Bis (dimethylethyl)-Phenol, Ethoxy ether butane, and Octadecenoic acid but the amountvaried (Table 3). However, apart from pesticidal activity, a few volatile compounds are also reported to possess antimicrobial activity, *i*.*e*., Hexadecane, Octadecene and Docosenamide [33, 34]. Hexadecene and Octosenamide were detected in *B*. *bassiana* strains, but Docosenamide was observed only in Bb6. Bb5 and Bb8 comprised of Tetradecene, Pentadecanoic acid and octadecadienoic acid, proved to be antibacterial and antifungal [35, 36].

**Table 3:** Lists of volatile organic compounds (VOCs) of ethyl acetate of *B*. *bassiana* strains analyzed by GC-MS.

Sl. No.	RT* (min.)	RI**	Compounds	Molecular formula	Relative area*** (%)					
					Bb5	Bb6	Bb8	Bb12	Bb15	Bb21
1	4.22	621	Methylethyl acetate	C_9_H_18_O_3_	0.26±0.11	1.40±0.29	-	3.70±0.35	2.33±0.25	3.70±0.46
2	4.47	669	Ethoxy ether butane	C_6_H_14_O	0.75±0.15	3.76±0.38	3.04±0.57	9.21±0.24	6.40±0.92	9.21±1.18
3	4.65	707	Methyl-2-pentanol	C_6_H_14_O	-	-	2.47±0.32	-	-	-
4	5.86	772	Hexanone	C_6_H_12_O	-	-	0.94±0.10	-	-	-
5	10.55	884	Methylene-1,3-dioxolane	C_4_H_4_O_3_	-	-	1.59±0.18	-	-	-
6	39.67	891	4-Hydroxy-oxecin-2-one	C_6_H_12_O_2_	5.33±0.98	-	-	-	-	-
7	38.43	1390	Tetradecene	C_14_H_28_	1.10±0.32	-	-	-	-	-
8	42.44	1442	Bis(dimethylethyl)-phenol	C_14_H_22_O	1.98±0.37	5.79±1.01	3.70±0.95	6.15±1.17	4.69±0.13	3.8±0.85
9	44.36	1592	Hexadecene	C_16_H_32_	2.41±0.24	6.77±0.95	3.52±0.31	5.44±0.78	5.45±1.15	5.44±0.29
10	46.83	1704	Tridecyl-2-propenoate	C_16_H_30_O_2_	0.44±0.05	-	-	-	-	-
11	48.80	1792	Octadecene	C_18_H_36_	2.40±0.19	7.63±1.02	5.07±0.65	11.68±0.11	6.51±0.39	11.68±1.48
12	50.05	1872	Pentadecanoic acid	C_15_H_30_O_2_	0.44±0.05	-	-	-	-	-
13	50.69	1896	Nonadecene	C_19_H_38_	-	19.51±3.18	5.64±0.39	4.72±0.28	-	4.72±0.26
14	51.18	1940	Hexadecenoic acid	C_16_H_30_O_2_	0.70±0.14	-	-	-	-	-
15	51.45	1962	*n*-Hexadecanoic acid	C_16_H_31_O_2_	36.18±2.19	9.06±1.15	16.03±1.02	15.20±2.53	9.97±0.15	14.20±1.17
16	51.68	2128	Cycloeicosane	C_20_H_40_	-	6.50±0.63	-	10.55±1.19	5.63±0.28	10.55±2.79
17	52.04	2126	Phenanthrenol	C_14_H_10_O	-	-	-	-	15.85±1.50	-
18	52.47	2175	Octadecadienoic acid	C_18_H_32_O_2_	-	-	23.16±3.47	-	-	-
19	52.76	2182	Octadecenoic acid	C_18_H_34_O_2_	36.89±1.74	6.12±1.17	9.64±1.12	11.28±1.26	3.80±0.16	11.28±1.12
20	53.28	2188	Docosene	C_22_H_44_	-	-	3.54±0.63	8.81±0.62	4.30±0.30	7.90±0.96
21	53.62	2192	Octadecanoic acid	C_18_H_34_O_2_	7.51±1.08	3.33±0.84	3.69±0.76	6.16±1.15	-	6.16±0.34
22	53.69	2300	Tricosene	C_23_H_46_	-	-	-	-	2.96±0.06	-
23	54.50	2392	Tetracosene	C_24_H_48_	-	5.31±0.72	-	-	-	-
24	54.90	2526	Methylpyridazinone	C_6_H_7_N	-	-	-	-	3.67±0.93	-
25	55.89	2608	Docosanol	C_22_H_46_O	2.63±0.64	-	-	-	-	-
26	55.80	2627	Docosenamide	C_22_H_43_NO	-	11.48±1.34	-	-	-	-
27	56.57	2828	Pentatriacontene	C_35_H_70_	-	-	-	3.29±0.66	-	-
28	56.49	2866	Tetracosahexene	C_6_H_2_O	-	-	2.90±0.13	-	-	-
29	56.70	2923	Cyclotetracosane	C_24_H_48_	0.98±0.16	5.88±0.24	2.19±0.24	-	-	-

*RT = Retention time (min.) of each volatile compound eluted through HP 5MS column in GC-MS

**RI: GC retention indices relative to C_9_–C_23_ *n*-alkanes on the HP-5MS column

***RA: Relative Area

**Fig 5:**
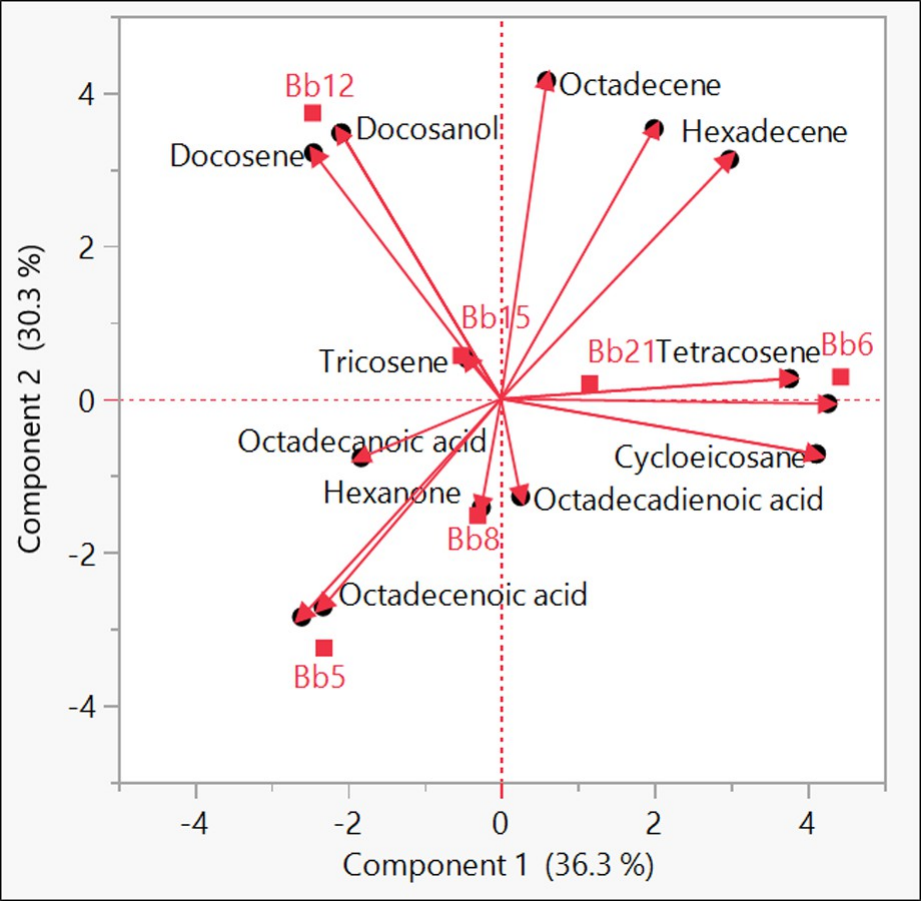
PCA biplot of bioactive compounds produced by six strains of *Beauveria bassiana*.

**Fig 6:**
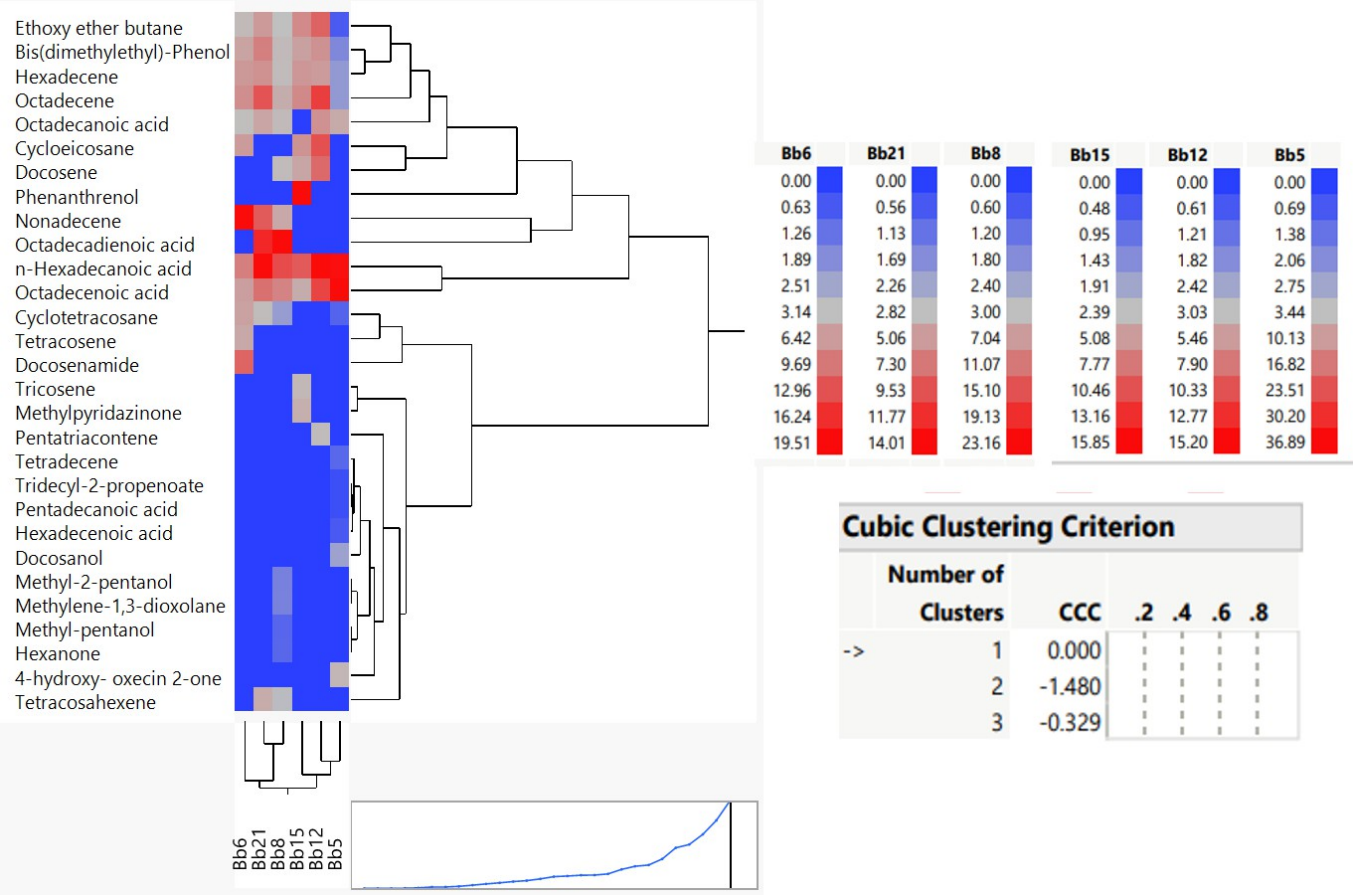
Heatmap and hierarchical clustering of GC-MS profiles of six strains of *Beauveria bassiana*.

Ramadan et al. suggest that PCA is a useful method for selectively finding the main influencing elements causing variations between samples [37]. It is employed in the current experiment to compare the visualisation and interpretation of the changes in the metabolite profiles of six *B*. *bassiana* strains. Fig 5 shows a PCA biplot for the ethyl acetate fraction of six *B*. *bassiana* strains. In the biplot, components 1 and 2 explained 36.3 and 30.3% of the variation, respectively. The results clearly differentiated the *B*. *bassiana* strains according to their potentiality against *T*. *truncatus*. Potential strains were separated into component 2, whereas non- potential strains were separated from potential strains along with component 1. We also showed distinct differences in the metabolomic profiles of potential and non-potential *B*. *bassiana* strains (Fig 6).

There was the least amount of similarity among the metabolomes of potential and non-potential strains (S7 Fig). Bis (dimethylethyl)-phenol was found to have acaricidal activity in the strains, but higher concentration,6.15%, 5.79% and 4.69% in the potential strains *i*.*e*., Bb12, Bb6 and Bb15 respectively. The same strains also exhibited higher pathogenicity toward *T*. *truncatus* during *in vitro* and green house studies. These findings clearly prove the potential effect of the volatile metabolites of *B*. *bassiana* strains against *T*. *truncates*, positively correlating with their intraspecific comprehensive metabolomic profiles.

#### Establishment of a dendrogram based on TLC profile

**Fig 7:**
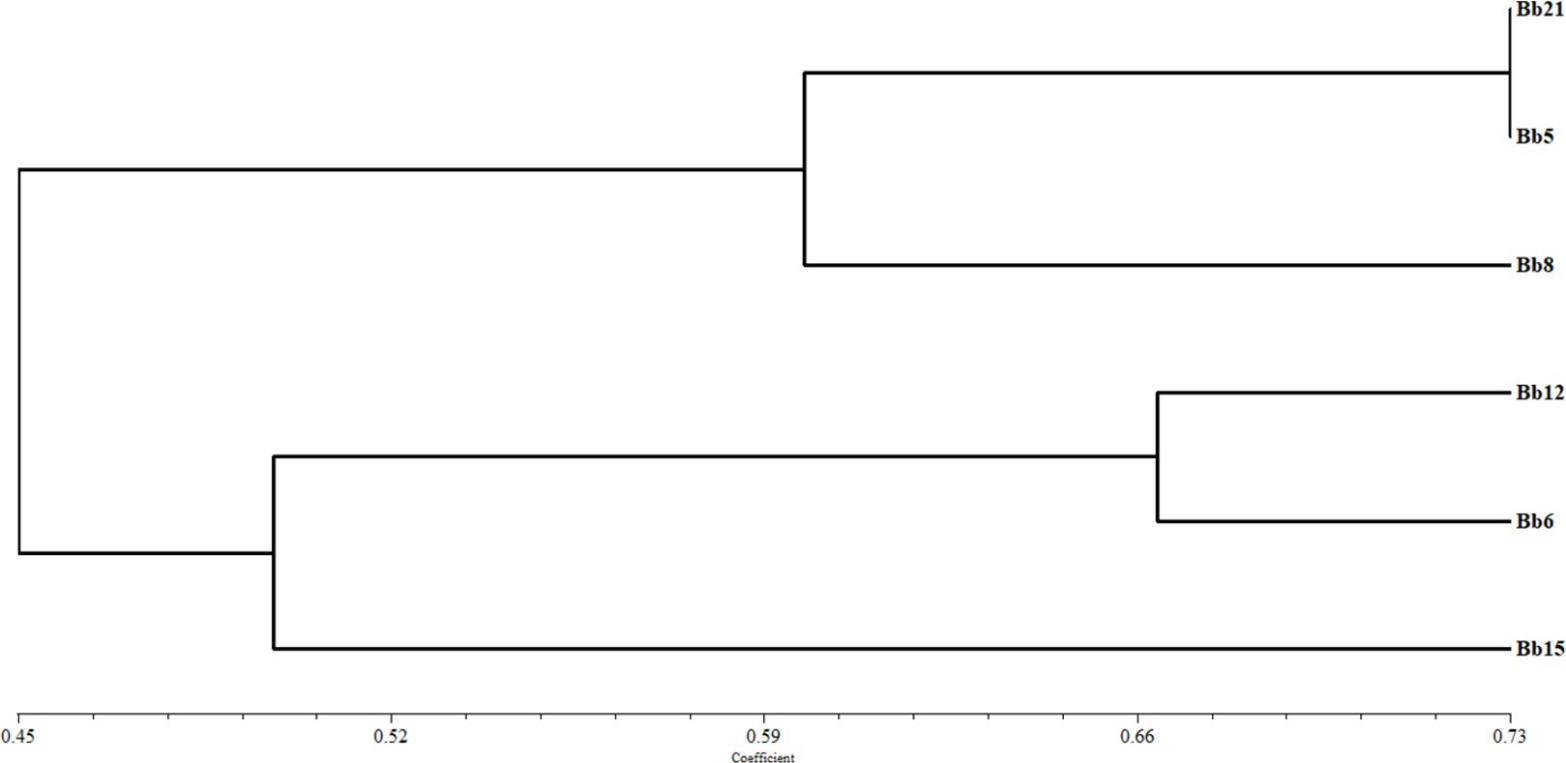
Dendrogram of six *B*. *bassiana* strains based on bioactive compounds using Thin layer chromatography.

Under UV light at 350 nm, each ethyl acetate fraction of the six tested strains of *B*. *bassiana* displayed a unique banding pattern (S4 Fig). Using the UPGMA (unweighted pair group method with arithmetic mean) method in NTSYSpc- 2.02e, dendrogram analyses were performed based on the banding patterns of *B*. *bassiana* metabolomes (S8 Fig). Two clades, Bb8, Bb5, and Bb21 (non-potential strains) forming one, and Bb6, Bb12, and Bb15 (potential strains) forming the other, were seen in the dendrogram (Fig 7). The results thus supported the phenomenon of positive correlation among the motabolomic profiles of potential and non- potential strains of *B*. *bassiana*, responsible for acaricidal action against *T*. *truncatus*.

## Discussion

Our preliminary proof of concept displayed that the primary bioassay of thirty *Beauveria* strains revealed a different rate of death and mycosis against *T*. *truncatus*. Out of 30 *Beauveria* strains, 6 strains (Bb5, Bb6, Bb8, Bb12, Bb15, and Bb21) were chosen for the current investigation in order to test their pathogenicity against *T*. *truncatus* under *in vitro* and greenhouse conditions to determine their LC_50_ and LC_90_ values using various doses of *B*. *bassiana*.

Research on fungal taxonomic classification has greatly benefited from the development of PCR amplification from various rDNA and TEF regions. All six *B*. *bassiana* strains taxonomic identities were established by alignments and phylogenetic studies. *B*. *bassiana* genetic diversity has been determined via analysis of ITS-rDNA sequences [38]). Using the ITS rDNA region [39, 40] evaluated the genetic diversity and phylogeny of five strains of *B*. *bassiana* (API 145, API 148, API 223, API 225 and API 226). Similarly, a recent DNA sequence analysis of *B*. *bassiana* ITS and elongation factor 1-(EF1-) genes demonstrated that sampling of widely dispersed species complexes in conjunction with morphological and molecular phylogenetic analysis is an effective strategy for assessing species diversity and the necessary first step in highlighting the species’ evolutionary history and historical ecology. *T*. *truncatus* is a severe pest of crops due to its ability to develop resistance to chemical pesticides, but little study has been done on microbial management as a substitute for chemical treatments [41]. This is the first study to use *B*. *bassiana* strains against *T*. *truncatus*. Despite the fact that previously described *B*. *bassiana*, strains were extremely pathogenic to insects [42]. Based on the pathogenicity test of thirty *B*. *bassiana* strains against *T*. *truncatus* by the leaf disc method under laboratory conditions, we chose the 6 *B*. *bassiana* strains from the previous work based on their mortality and mycosis rate against *T*. *truncatus*. Therefore, we believed that mortality percentage and the quality and quantity of the metabolic compounds were the best indicators to prove the ability of *B*. *bassiana* strains to manage *T*. *truncatus* populations.

The effectiveness of six *B*. *bassiana* strains against *T*. *truncatus* was assessed in both leaf discs and potted plants of *Phaseolus vulgaris* Linnaeus (Fabales: Fabaceae). [43], found that the fungal strains and conidial concentrations had different mortality rates for insects. According to the results of the current study, the strains Bb6, Bb12, and Bb15 were proved to be effective BCAs against *T*. *truncatus* because they caused the highest mortality rates, *viz*., 95.67%, 90.06%, and 93.45%, respectively, at 3 DAI and higher mycosis (sporulation) rates, *viz*., 88%, 84%, and 88%, respectively, at 5 DAI than the other strains. These findings are parallel to the observation of [44], where, in 10 DAI of *B*. *bassiana* application, 73.1% of *T*. *urticae* mites perished. On the seventh day, *B*. *bassiana* was found to be effective against *T*. *urticae*, causing 63.31 percent mite mortality at a concentration of 0.3×10^8^ conidia/mL [45]. In another study [46], found that the pathogenicity effects of the two different strains of *B*. *bassiana* on *T*. *urticae* were 24–60% on the third day, 62.7–88% on the fifth day, and 90.7–100% on the seventh day. According to [47], the percentage of *T*. *urticae* deaths ranged from 55 to 82%.

A bioassay was also done on potted bean plants to imitate the potential efficacy of 6 *B*. *bassiana* strains under semi-field circumstances. Plants sprayed with 1×10^8^ conidia/mL of the Bb6, Bb12, and Bb15 strains showed a lower number of *T*. *truncatus* populations at 48 HAS than the control plants. The present study’s results are consistent with those of other researchers who have utilized entomopathogenic fungi to control mites [48]. [49] investigated the pathogenicity of strains of *B*. *bassiana* against *T*. *urticae* adults and discovered that mite mortality caused by fungus at 72 h post-inoculation than that of the negative control (P 0.05).These three high potential strains were also tested against *T*. *truncatus* at various conidial concentrations (10^6^–10^9^ conidia/mL). At 3DAI, the LC_50_ values of the Bb6, Bb12, and Bb15 strains were 1.4×10^6^, 1.7×10^6^ and 1.4×10^6^ respectively. The LC_90_ values of the Bb6, Bb12, and Bb15 strains were 7.3×10^7^, 1.4×10^8^ and 4.2×10^8^ respectively. These are consistent with past studies [50], who found that *B*. *bassiana* concentrations of 1×10^5^–1×10^8^ conidia/ml applied to *T*. *urticae* adults exhibited 42–64% mortality of mites at the LC_50_ of 0.3 × 10^8^ conidia/mL. According to [51], *B*. *bassiana* induced 15–70% mortality against *T*. *urticae*, with its LC_50_ and LC_90_ values reported as 3.3 × 10^6^ and 7.8 × 10^9^ conidia/mL, respectively. Additionally, it was also reported that between 56.4 and 82.6% of *T*. *evansi* were killed by *B*. *bassiana* on the 7th day, with an LC50 value of 1.1 ×10^7^ conidia/mL [52].

The different *B*. *bassiana* strains exhibit varying degrees of virulence against *T*. *truncatus*, which may be related to the enzymes formed by each strain. [53] hypothesised that the presence of enzymes that affect the fungus penetration process is likely responsible for the variations in the virulence of entomopathogenic fungal strains. The observed variation in virulence may potentially be due to secondary metabolites produced by entomopathogen fungi, such as beauvericin, a toxin found in *B*. *bassiana*. Therefore, in order to understand the relationship between metabolite profiling and their pathogenicity against *T*. *truncatus*, we continued our analysis of the metabolite profiles of potential and non- potential intraspecific *B*. *bassiana* strains. In order to examine the heterogeneity in the metabolome profiles of three strains of *B*. *bassiana*, MH590235 (TM), MK918495 (BR), and KX263275 (BbI8) [54], employed GC-MS analysis. The metabolite profile differed within the species, and distinct profiles were discovered in the three research strains, TM, BR, and BbI8. [55] discovered the presence of alcohols, amino acids, organic acids, phosphoric acids, purine nucleotides and bases, sugars, saturated fatty acids, unsaturated fatty acids, or fatty amides in extracts of Cordyceps bassiana fruiting bodies that were 70% methanol and 100% hexane. The potential strains *i*.*e*., Bb6 (5.79%), Bb12 (6.15%), and Bb15 (4.69%) had higher levels of acaricidal metabolites (Bis(dimethylethyl)-phenol) than non-potential strains Bb5 (1.98%), Bb8 (3.70%), and Bb21 (3.8%), according to GC-MS analysis of ethyl acetate fractions. Insects also showed an immune response against *B*. *bassiana*, which can be counteracted through the impacts of secretion or excretion products that interfere with and break the cells and molecules of innate immunity. Likewise, apart from acaricidal, Bb6 and Bb12 potential strains have higher amounts of defense secretion compound, *i*.*e*., Nonadecene (19.51% and 4.72% respectively), which may help to increase their pathogenicity by overcoming the insect innate immunity. Any single metabolite may not be dependable for the increased virulence of the potential strains of *B*. *bassiana*. As a result, the potential *B*. *bassiana* strains contain mite specific acaricidal compound (Bis(dimethylethyl)-phenol), insecticidal (*n*-Hexadecanoic acid) and insect innate immunity overcoming compound (Nonadecene); thus the synergistic effect of these volatile compounds may result r in pathogenicity against *T*. *truncatus*. However, in the TLC analysis, we observed that the potential and non-potential strains formed two different clusters in the dendrogram. We may thus draw the conclusion from these findings that there is a correlation between the metabolite profiles and the potential of intraspecific *B*. *bassiana* strains against *T*. *truncatus*.

## Conclusion

Confirm the efficacy of 6 local strains of *B*. *bassiana* against *T*. *truncatus* both in leaf discs and in potted plants of *Phaseolus vulgaris*. Bb6, Bb12 and Bb15 strains exhibited a higher mortality rate that exceeded 50% at lower concentrations than the non-potential strains. Also,based on metabolomic studies, there is a correlation between the metabolite profile of six strains of *B*. *bassiana* and their potentiality against *T*. *truncatus*. So far, there are no reports on the study of bipartite interactions of molecular pathogenicity and metabolomics of *B*. *bassiana* against *T*. *truncatus* and thus, the study’s findings can be used to determine the pathogenicity genes and their metabolites of these strains during the infection of *T*. *truncatus*. After the confirmation of the field studies, these potential strains can be used as biological control agents.

## Supporting information

S1 Fig GC-MS chromatogram of secondary metabolites from Bb5 strain of *B*. *bassiana*. (TIF)

S2 Fig GC-MS chromatogram of secondary metabolites from Bb6 strain of *B*. *bassiana*. (TIF)

S3 Fig GC-MS chromatogram of secondary metabolites from Bb8 strain of *B*. *bassiana*. (TIF)

S4 Fig GC-MS chromatogram of secondary metabolites from Bb12 strain of *B*. *bassiana*. (TIF)

S5 Fig GC-MS chromatogram of secondary metabolites from Bb15 strain of *B*. *bassiana*. (TIF)

S6 Fig GC-MS chromatogram of secondary metabolites from Bb21 strain of *B*. *bassiana*. (TIF)

S7 Fig Venn diagram representing the GC-MS profile of potential and non-potential strains of *B*. *bassiana*. a) Potential strains: Bb6, Bb12 and Bb15; b) Non-potential strains: Bb5, Bb8 and Bb21.
(TIF)

S8 Fig Thin Layer Chromatography of six *B*. *bassiana* strains. Mobile phase was Toulene, ethyl acetate, and formic acid (5:4:1).
(TIF)

S1 Table Corrected mortality and mycosis of *Tetranychus truncatus* infected by *B*. *bassiana*. (PDF)
