# Supplementary figures and images for: “Metabolomic diversity of local strains of Beauveria bassiana (Balsamo) Vuillemin and their efficacy against the cassava mite, Tetranychus truncatus Ehara (Acari: Tetranychidae)”
Source: PLoS One. 2022 Nov 15;17(11):e0277124. doi: 10.1371/journal.pone.0277124 (PMC9665378; doi:10.1371/journal.pone.0277124)

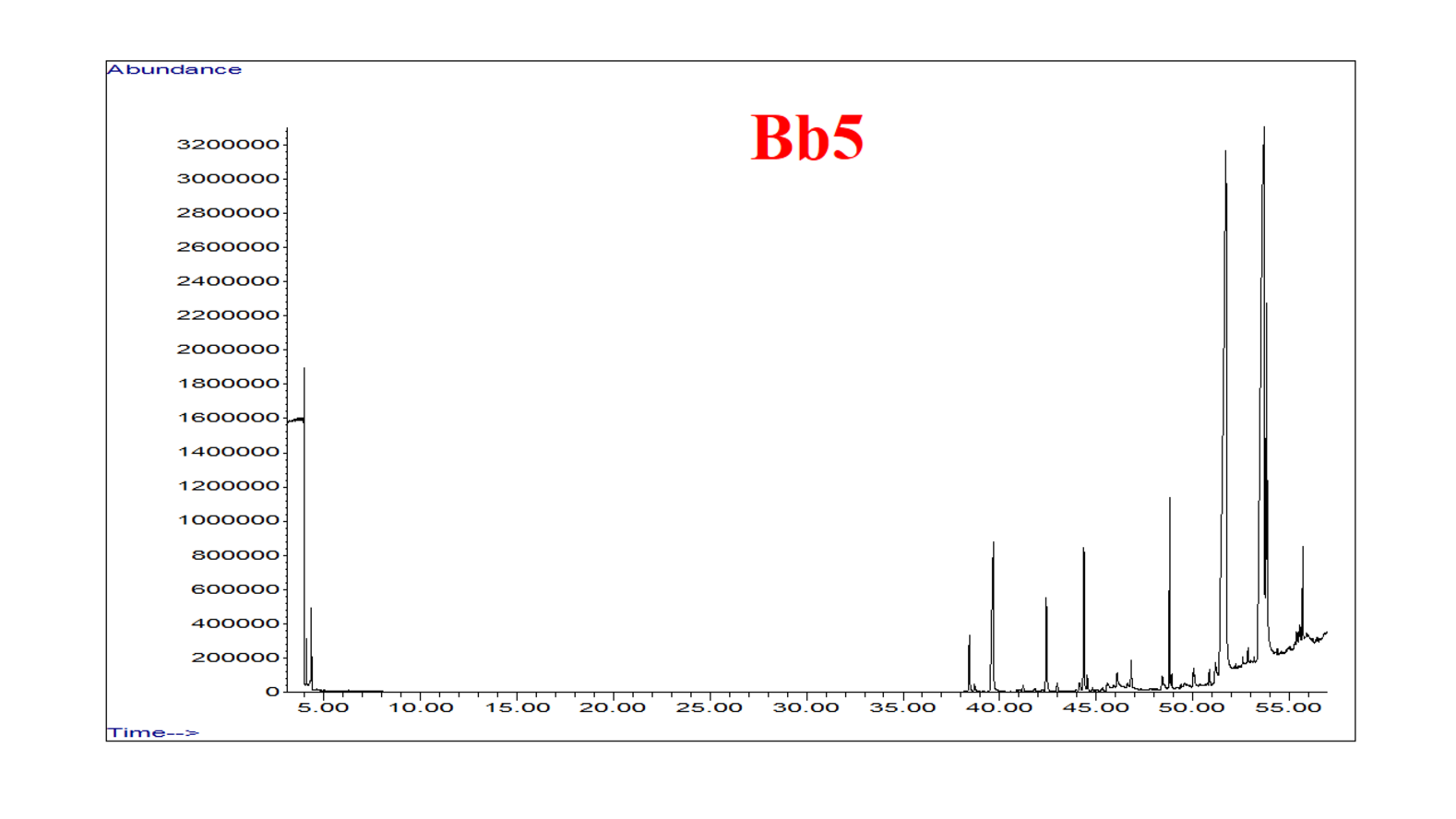

Supplement: S1 Fig — (TIF) [file pone.0277124.s001.tif]

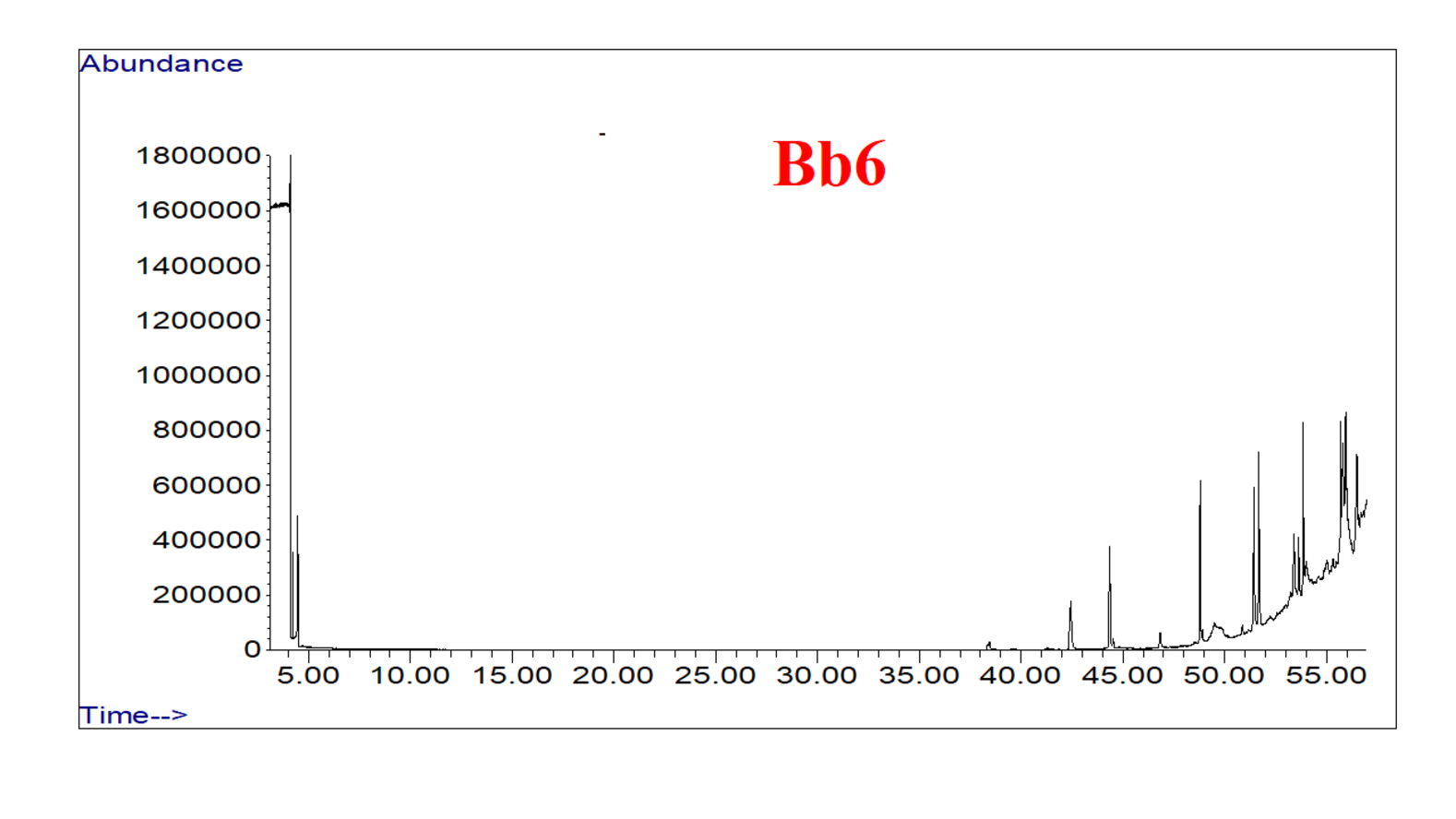

Supplement: S2 Fig — (TIF) [file pone.0277124.s002.tif]

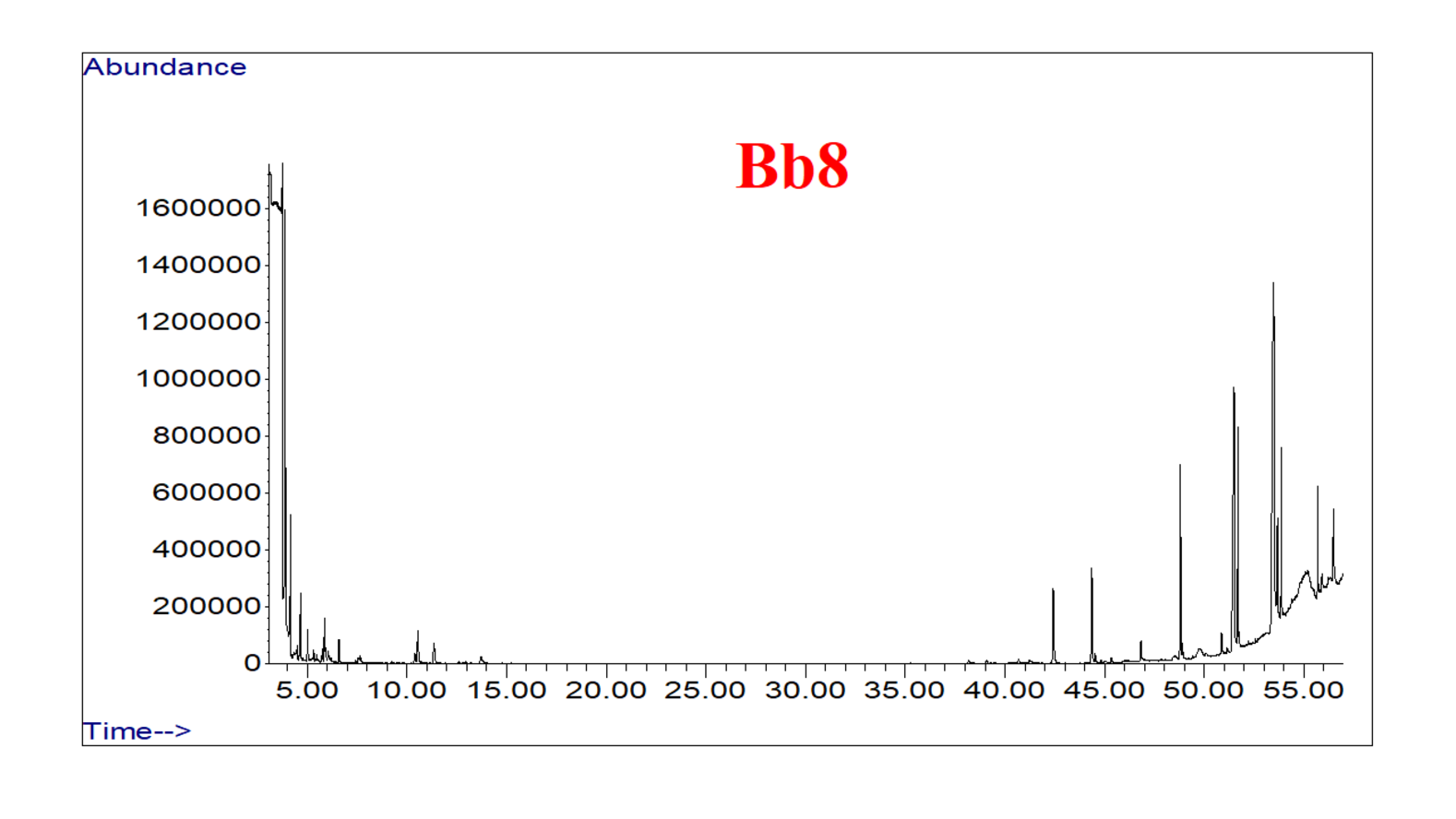

Supplement: S3 Fig — (TIF) [file pone.0277124.s003.tif]

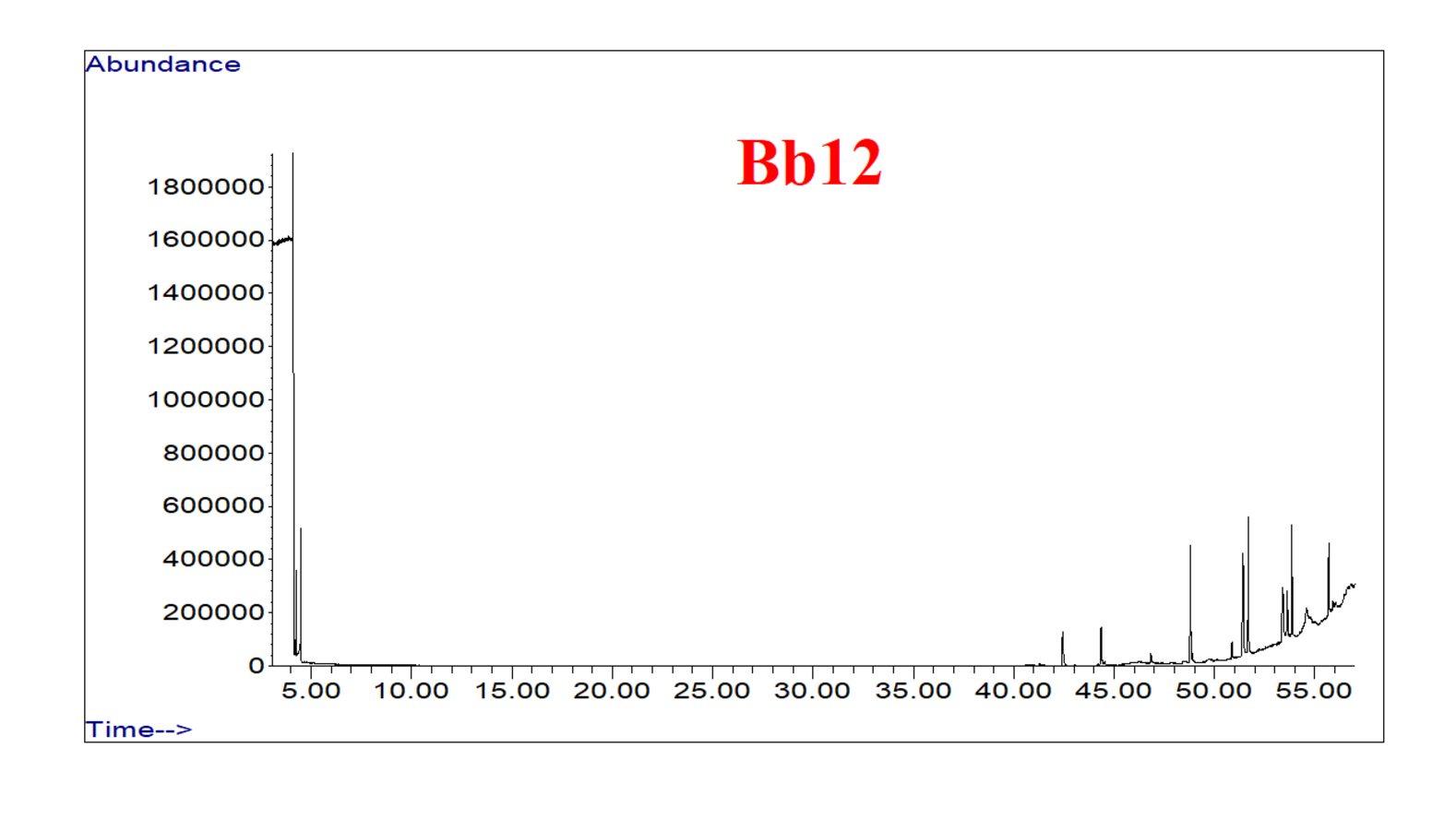

Supplement: S4 Fig — (TIF) [file pone.0277124.s004.tif]

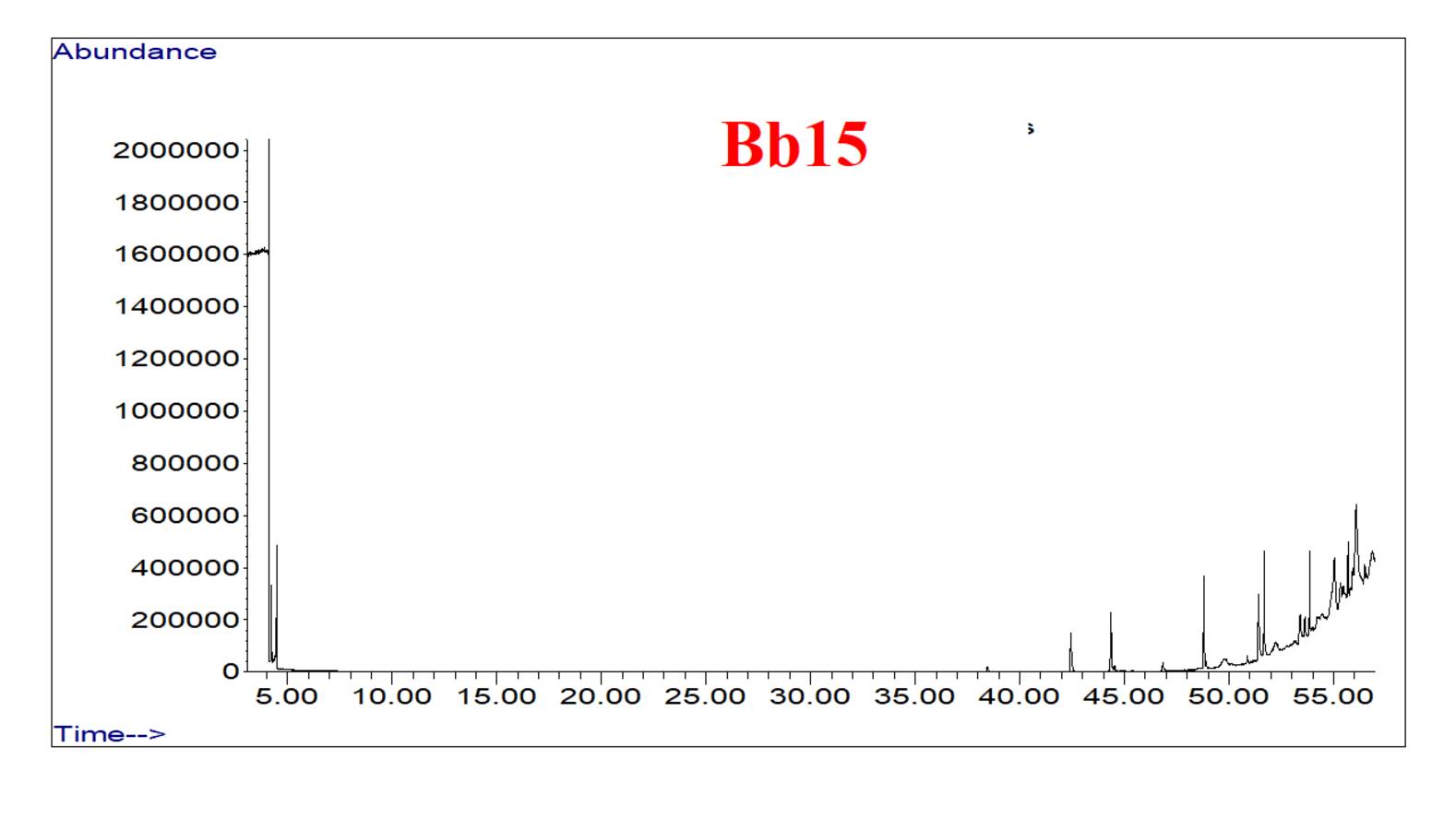

Supplement: S5 Fig — (TIF) [file pone.0277124.s005.tif]

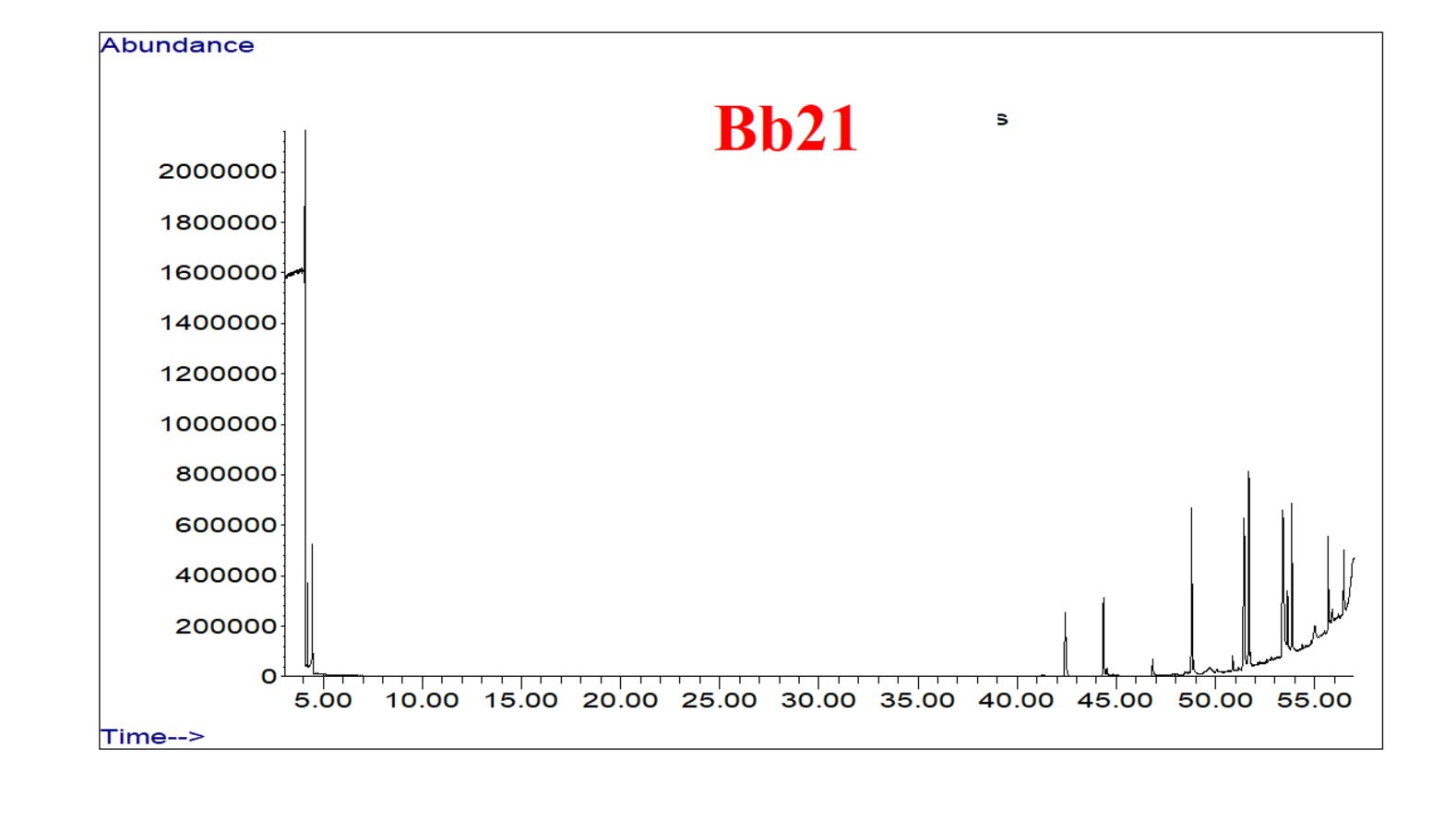

Supplement: S6 Fig — (TIF) [file pone.0277124.s006.tif]

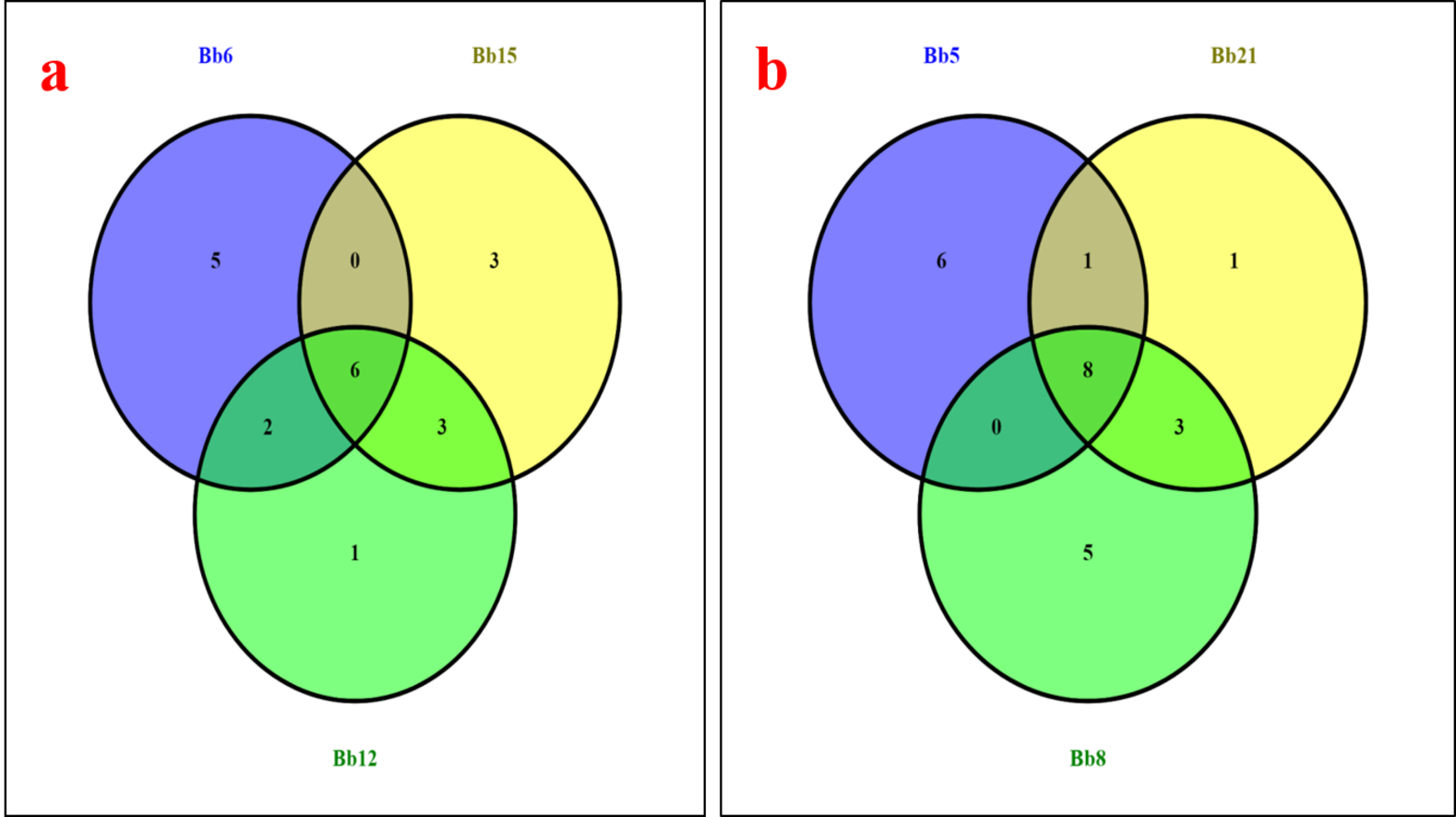

Supplement: S7 Fig — a) Potential strains: Bb6, Bb12 and Bb15; b) Non-potential strains: Bb5, Bb8 and Bb21. (TIF) [file pone.0277124.s007.tif]

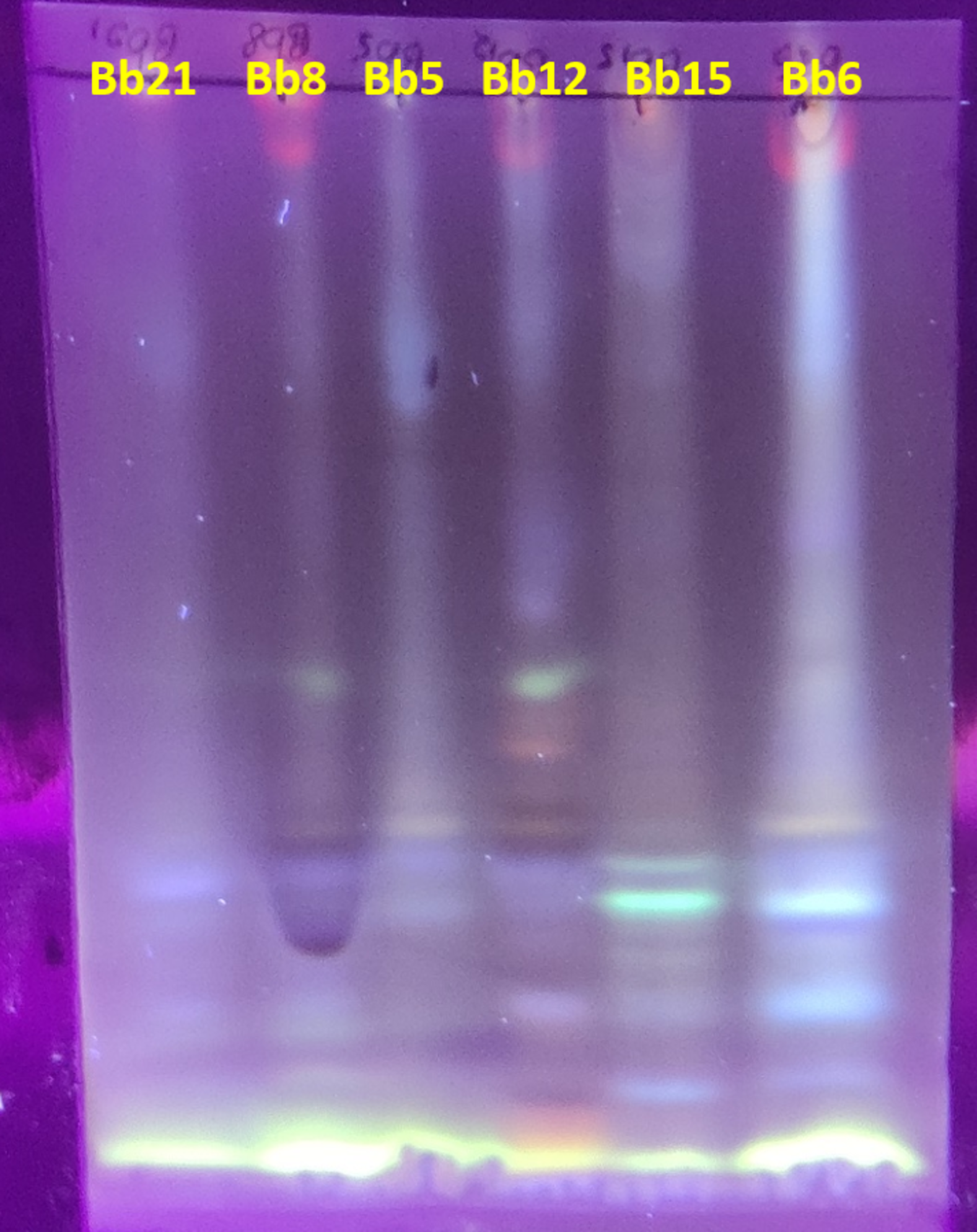

Supplement: S8 Fig — Mobile phase was Toulene, ethyl acetate, and formic acid (5:4:1). (TIF) [file pone.0277124.s008.tif]
