# Supplementary material for: “Metabolomic diversity of local strains of Beauveria bassiana (Balsamo) Vuillemin and their efficacy against the cassava mite, Tetranychus truncatus Ehara (Acari: Tetranychidae)”
Source: PLoS One. 2022 Nov 15;17(11):e0277124. doi: 10.1371/journal.pone.0277124 (PMC9665378; doi:10.1371/journal.pone.0277124)
Supplement: S1 Table — (PDF) [file pone.0277124.s009.pdf]

S1 Table: Corrected mortality and mycosis of *Tetranychus truncatus* infected by *B. bassiana*.

Isolates	*% Corrected mortality mean ± SEM	% Mycosis mean ± SEM
	**7 DAI**	**8 DAI**
Bb1	70.70 (56.53)± 1.55ef	50 (44.99)± 1.58jk
Bb2	56.49 (47.78)± 1.38d	17 (24.12) ±1.96g
Bb3	65.26 (53.94) ±1.55def	52 (46.15) ±1.47jk
Bb4	28.13 (31.93) ±1.78bc	15 (22.67) ±1.29fg
Bb5	11.93 (19.91) ±1.88a	0 (0.00) ±0.00a
BB6	97.78 (84.55) ±3.34i	88 (70.06) ±1.95no
Bb7	74.85 (60.08) ±2.06fg	53 (46.73) ±2.16jkl
Bb8	6.49 (13.06) ±3.51a	1 (2.58) ±2.58ab
Bb9	67.31 (55.20) ±1.63def	42 (40.37) ±1.48ij
Bb10	34.74 (36.06) ±1.55bc	14 (21.46) ±2.61fg
Bb11	41.35 (40.00) ±1.34c	31 (33.70) ±2.10hi
Bb12	94.50 (79.56) ±4.41i	85 (67.68) ±2.50no
Bb13	77.08 (61.51) ±1.57fg	69 (56.29) ±2.22m
Bb14	23.86 (29.11) ±1.77b	11(19.07) ±1.82ef
Bb15	96.73 (81.90) ±3.31i	88 (71.89) ±4.71o
Bb16	32.57 (34.75) ±1.40bc	11(17.24) ±4.57def
Bb17	70.64 (57.26) ±1.41ef	68 (55.74) ±2.56m
Bb18	31.40 (33.99) ±1.78bc	6 (9.00) ±5.66bcj
Bb19	69.59 (56.62) ±1.71ef	51 (45.60) ±2.13k
Bb20	76.02 (60.80) ±1.60fg	66 (54.39) ±1.74l
Bb21	26.37 (29.55) ±8.14b	5 (9.73) ±4.36bcd
Bb22	35.85 (36.74) ±1.26bc	12 (19.83) ±2.30ef
Bb23	24.97 (29.84) ±1.82b	7 (11.93) ±4.94cde
Bb24	89.12 (71.59) ±3.10h	81 (64.33) ±1.77mno
Bb25	58.71 (50.02) ±0.62de	23 (28.58) ±1.33gh
Bb26	88.01 (70.07) ±1.95h	78 (62.29) ±2.35m
Bb27	83.63 (66.62) ±2.66gh	77 (61.49) ±1.78mn
Bb28	83.68 (66.30) ±1.38gh	71(57.54) ±1.88m
Bb29	77.13 (61.46) ±0.84fg	64 (53.18) ±1.47kl
Bb30	85.91(68.27) ±2.03gh	76 (61.08) ±2.92n
Beveroz	74.75 (60.04) ±2.02fg	52 (45.73) ±2.15jkl
F ratio	54.16	66.66

Figures in parentheses are arcsine transformation values.

*Means followed by the same letter within the same column are not significantly different ($p<0.05$).
